# How health systems factors, socioeconomic status, and care-seeking practices influence the cost of rotavirus-related diarrhea care in Mukuru informal settlements, Kenya

**DOI:** 10.3389/fpubh.2026.1828318

**Published:** 2026-07-24

**Authors:** Shalon Gatwiri Njue, Mercy G. Mugo, Evans Kipngetich Kibet, James Kariuki, Evans Kiptanui, Cecilia Mbae, Phelgona Otieno, Samuel Kariuki, Fred Were

**Affiliations:** 1 Centre for Microbiology Research, Kenya Medical Research Institute, Nairobi, Kenya; 2 Department of Economics and Development Studies, University of Nairobi, Nairobi, Kenya; 3 Centre for Public Health Research, Kenya Medical Research Institute, Nairobi, Kenya; 4 Centre for Clinical Research, Kenya Medical Research Institute, Nairobi, Kenya; 5 Department of Pediatrics and Child Health, University of Nairobi, Nairobi, Kenya

**Keywords:** cost of illness, economic burden, health seeking behavior, out of pocket expenditure, rotavirus, social economic determinants of health, under five children, urban under-resourced areas

## Abstract

**Background:**

Diarrheal diseases remain a leading cause of death, especially in Africa, accounting for 1.17 million deaths globally in 2021, with rotavirus responsible for 32.2% of these deaths. Since the introduction of rotavirus vaccines in Kenya in 2014, several studies have demonstrated the clinical impact of the vaccine, showing a reduction in child deaths from diarrhea. However, limited evidence exists on the socioeconomic burden that is still posed by rotavirus diarrhea, especially in Kenya, where the prevalence remains high at 14%, as reported in the 2022 Kenya Demographic and Health Survey. The persistence of diarrheal burden and hospitalizations despite investments in diarrheal prevention and control suggests that medical and public health interventions do not automatically reduce the health burden and related economic burden and require the integration of policies that address other socioeconomic determinants of health demand. This study sought to investigate how health-seeking patterns and socioeconomic status intersect and influence the cost burden of diarrheal illnesses in Mukuru informal settlements in Kenya.

**Methods:**

A cross-sectional study was conducted between May 2023 and July 2025 among caregivers of children under five presenting with diarrhea at Mukuru clinics. Of the 500 diarrheal cases, 100 laboratory-confirmed rotavirus cases were analyzed. Costs were categorized as direct medical, direct non-medical, and indirect costs. Descriptive and stratified analyses were used to explore cost variations by socioeconomic status and care-seeking behavior. A gamma regression model assessed associations between the cost of illness and predictors including education, employment, prior care-seeking, and type of facility visited.

**Results:**

The average cost of treating a diarrheal episode was estimated at $8.88 (95% CI: 7.25, 10.51) per patient. Direct medical costs comprised 60.36% of this cost and were mainly driven by drug prescriptions purchased outside clinics (30.18%), expenditure at other facilities on self-medication (19.37%), and expenditure at Mukuru clinics (10.81%). Income lost through work absence or leisure accounted for 36.82% at an average cost of $3.17 (95%:2.95, 3.58). Fragmented care pathways, drug stockouts, delayed care seeking, and inappropriate self-medication significantly increased household costs, regardless of insurance status. Employment status and type strongly influenced indirect costs, aggravated by long waiting times and repeated care-seeking.

**Conclusion:**

Overall, the findings indicate that the cost of childhood diarrhea in Mukuru is driven less by caregiver socioeconomic characteristics—given that Mukuru residents share similar vulnerabilities—and more by health-seeking behavior and health system factors, particularly drug availability and fragmented care pathways. Strengthening drug supply chains, improving the quality and reliability of public primary healthcare, and promoting timely and proper care-seeking pathways could substantially reduce household costs and improve financial protection in urban informal settlements.

## 1 Introduction

Diarrheal diseases have remained a leading cause of death, especially in Africa, according to the World Health Statistics 2024 (1). Rotavirus is a diarrheal pathogen spread predominantly by the fecal-oral route through contamination of food, water, and environmental surfaces. According to the Lancet Global Burden of Disease 2021 report, diarrhea caused approximately 1.17 million deaths globally in 2021, and rotavirus was a predominant pathogen causing 32.2% of diarrheal mortality in children under 5 years of age (2). In Kenya, the prevalence of diarrhea among children under 5 years of age is estimated at 14% according to the 2022 Kenya Demographic and Health Survey (KDHS).

Children living in urban informal settlements remain at greater risk of diarrhea and death (3). Mukuru informal settlement is a under-resourced area located approximately 20 km from Nairobi city, characterized by large populations overcrowded in tiny spaces, poor structures, and open sewer drains, which greatly contribute to the risk of enteric infections. Poverty is also widespread in Mukuru, and the majority of residents survive on informal and unstable employment, further exacerbating vulnerability to catastrophic expenditures (4, 5). Diarrhea is highly prevalent in Mukuru and is caused by a broad range of diarrheal pathogens, not limited to *E. coli*, Salmonella, and typhoid (6).

Since Kenya’s introduction of rotavirus vaccines in 2014, there has been a reduction in child deaths from rotavirus diarrhea, with a prevalence of 14.5% in 2018 post-vaccine introduction, which is lower than the prevalence of 38.2% observed in 2008 pre-vaccine introduction (7, 8). However, significant cases of severe rotavirus diarrhea episodes, hospitalizations, and deaths continue to be detected post-vaccine introduction in Nairobi (9–11). In 2010 and 2014, the Kenyan Ministry of Health formulated policies and guidelines to control diarrheal diseases, which addressed access to treatment, including improving the supply chain of zinc and ORS, handwashing, vitamin A supplements, and the introduction of rotavirus vaccines into the national immunization schedule (12, 13). Despite these efforts, the prevalence of diarrhea remains at 14%, as reported in the 2022 Kenya Demographic Health Survey (14). Previous studies have evaluated the implementation of these national policies and guidelines and identified persistent gaps, including a limited focus on socioeconomic determinants of health, environmental and zoonotic transmission pathways, weak funding structures, and demographic and other contextual drivers of diarrhea (15). In addition, suboptimal vaccine uptake continues to constrain effective diarrheal disease prevention efforts. In 2024, the national coverage of the last rotavirus vaccine dose was estimated to be 82% (16), reflecting gaps in vaccine uptake. Barriers to vaccination include caregivers’ lack of awareness of vaccine benefits, safety concerns, and socioeconomic constraints that shape health-seeking behaviors (17, 18). Health-seeking behaviors directly influence both clinical outcomes and household economic burden. Caregivers may delay or forgo formal care because of distance, cost, or lack of knowledge, often resulting in higher medical expenses and productivity losses (2, 17, 19–23).

Apart from the health burden, rotavirus infection may lead to impoverishment of households owing to treatment expenses and loss of productivity. Previous studies have demonstrated that low-income households spend a significant proportion of their income on treatment and hospitalization for rotavirus diarrhea (10, 24–28). However, there is still limited evidence on the socioeconomic burden of rotavirus diarrhea, especially in informal settlements where rotavirus remains a major cause of pediatric diarrhea. Existing studies have demonstrated that income levels affect care decisions for children with diarrhea (29) and that appropriate health-seeking practices for childhood diarrhea remain a significant challenge among urban informal settlements in Kenya (30). There are also gaps in the economic burden of diarrheal disease in the Kenyan context and in how health-seeking patterns and socioeconomic status intersect to influence the cost of diarrheal illnesses. Evidence from other LMICs indicates that the mean cost per diarrhea episode ranges between $4.30 and $145.47 per outpatient episode and between $41.01 and $538.33 per inpatient episode (27, 31, 32).

The persistence of diarrheal burden and hospitalizations despite investments in diarrheal prevention and control suggests that medical and public health interventions do not automatically translate to reduced economic and health burden and require integration with policies that address other socioeconomic determinants of health demand. To address these evidence gaps, this study assessed the cost of diarrheal diseases among children under five living in Mukuru informal settlements to provide cost evidence on the burden of diarrhea, especially from a household perspective, and to examine how health-seeking patterns and socioeconomic status influence the cost. The findings from this study may inform the Mukuru community on the benefits of timely and efficient care-seeking practices and policymakers on how to strengthen financial risk protection mechanisms and promote equitable diarrhea care in low-resource settings such as Mukuru.

## 2 Methods

### 2.1 Study sites

This study was conducted in Mukuru informal settlements in Nairobi, Kenya. Mukuru is an urban informal settlement located on the southeastern side of Nairobi, 20 km from Nairobi’s central business district. Four facilities, Mukuru Health Centre (MCC), a public health facility; Medical Missionaries of Mary (MMM), a private hospital; Mukuru Reuben Health Centre, a public hospital; and Lady of Nazareth Mukuru Njenga Hospital, a public hospital, from two of the largest villages, Mukuru kwa Njenga and Mukuru kwa Reuben, which have a combined population of 385,000, were mapped for surveillance. The facilities included a mix of private and public hospitals, and caregivers from Mukuru village presenting children with diarrhea at these facilities were interviewed. Mukuru has a dense population, with approximately 70,000 people living in an area of 2.3 km^2^. The village is characterized by widespread poverty, houses without spacing and ventilation, with each housing approximately five occupants (33). These environmental and hygiene conditions put Mukuru children at a high risk for diarrheal infections (34).

### 2.2 Study design and sampling

This study adopted a cross-sectional design to recruit children under five presenting with over three diarrhea episodes.

The Kenya Bureau of Statistics 2019 census report estimated the number of children under five in Kenya at 5.99 million, out of a total population of 47.6 million, which is approximately 12%. The census also established that Mukuru kwa Reuben and Mukuru kwa Njenga had a total population of 385,056. Based on statistics, the population below the age of 5 in Mukuru is approximately 46,207.

Sample size was calculated using Fisher’s method, which was appropriate for cross-sectional studies aimed at estimating the economic burden among a defined population at a single point in time (35–37).


$$n=\frac{Z^{2}*P*(1-P)}{d^{2}}$$

Where:

- *n* is the minimum sample size,
- Z is the standard deviation at 95% with a confidence interval of 1.96, and
- P = the approximate estimate of diarrhea disease prevalence, which is 14% according to the 2022 KHDS, and
- d is the error margin of 5%.

$$n=\frac{1.96^{2}*0.14*(1-0.14)}{0.05^{2}}=185\;\text{respondents}$$

Inclusion criteria were children under 5 years of age presenting with acute diarrhea, i.e., at least three loose stools in the preceding 24 h at Mukuru clinics. Clinicians helped identify children with diarrhea. Once a child was identified, the study team invited caregivers to participate upon providing informed consent. While the required sample was 185, over 500 caregivers were recruited, and using a structured cost questionnaire, they provided their socioeconomic characteristics, health-seeking patterns, and costs incurred, while the clinical team collected stool samples and rectal swabs. The laboratory team tested the stool samples using TaqMan array card PCR. Of the 500 diarrheal samples screened, 100 tested positive for rotavirus and formed the final analytical sample for this study.

### 2.3 Costing methods

The study adopted a household perspective to estimate the economic burden of rotavirus diarrhea faced by families in Mukuru. This perspective captured household expenses that consist mainly of out-of-pocket expenses attributed to the treatment process and productivity losses associated with time taken by caregivers of children under 5 years to seek health care.

Costs were categorized into direct medical costs, direct non-medical costs, and indirect costs. Direct medical costs comprised drugs, registration fees, and consultation fees incurred before or during seeking treatment at the clinic. Direct non-medical costs included incidentals spent while seeking healthcare, such as transport costs and meals. Indirect costs include productivity losses as a result of absence from work (38–45).

Questionnaires were used to estimate parent/caregiver direct costs of diarrheal disease and to understand patterns of healthcare-seeking behavior. Market surveys and hospital records were used to determine the costs of the prescribed drugs. Given that the study population comprised predominantly residents of under-resourced areas, many of whom were unemployed or engaged in informal employment, indirect costs were measured through productivity losses utilizing the Kenyan minimum wage guidelines. Minimum wage was used as a standardized measure across households to minimize potential bias associated with self-reported income estimates (46, 47).

These costs were categorized across several indicators to capture the influence of socioeconomic determinants and health-seeking behaviors on household expenditure. Socioeconomic status was described using caregiver education level and employment status, while health-seeking patterns were described based on prior care-seeking behavior, type of health facility visited, and insurance status. The total cost represented the aggregated sum of the direct medical, direct non-medical, and indirect costs.

### 2.4 Data management and analysis

Data were collected and stored using EpiCollect5, which ensured that data validation and cleaning were performed in real time throughout the data collection period while also enhancing efficiency and data security. Data were analyzed using Stata version 15 and Microsoft Excel.

A descriptive and stratified analysis was conducted to stratify and describe the costs according to socioeconomic status and health-seeking patterns. The costs were summarized as measures of central tendency using mean, median, and standard deviation. Patterns in spending across the various categories were checked to identify themes such as access to care and efficiency, ability or willingness to pay, and health system issues.

Cost data are generally categorized as non-negative integers, skewed to the right, and very variable, with several houses incurring low costs and a few incurring very high costs (48, 49). A gamma link regression model was therefore used to infer the relationship between the cost of illness, health-seeking patterns, and socio-economic status. The cost of illness was the dependent variable, and the predictor variables were education level, employment status, prior care seeking, and the type of health facilities visited.

**Figure 1:**
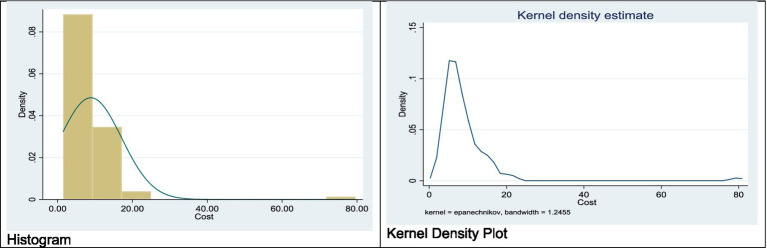
Distribution of total cost.

Prior to the model estimation, diagnostic tests were conducted to determine the distribution of the total cost (Table 1). Descriptive statistics indicated overdispersion, with variance greatly exceeding the mean. Formal tests further confirmed significant deviations from normality, as evidenced by the histogram and kernel density distribution (Figure 1), the Shapiro–Wilk and skewness kurtosis tests (*p* < 0.001). In addition, the Breusch–Pagan test indicated the presence of heteroscedasticity (*p* < 0.001), thereby violating the key assumptions of ordinary least squares (OLS) regression. Given the distributional properties of the data, a generalized linear model was considered appropriate, as it relaxes the assumptions of the OLS regression (50–53). The gamma family with a log link function was selected owing to its suitability for modeling continuous, positively skewed data, as is typical for health cost data. The log-link function ensured that the predictor coefficients remained positive and had a multiplicative effect.

**Table 1:** Model diagnostics.

Statistical test	Test results					
Dispersion of total cost	Observations	Mean	Std. Dev.	Variance	Min	Max
	100	8.88	8.22	66.84	1.5	79.6
Shapiro wilk test for normality	Observations	W	V	z	Prob>z	
	100	0.47	43.65	8.37	0	
Skewness/Kurtosis test	Observations	Pr (Skewness)	Pr (Kurtosis)	adj	chi^2^ (2)	Prob > chi^2^
	100	0.00	0.00			0.00
Breusch-Pagan test for heteroskedasticity	chi^2^(1)	Prob > chi^2^				
Constant variance	30.41	0.00				

Therefore, the model is specified as follows:

$\begin{array}{l} \mathrm{Log}(E[\text{cost of illness}])=B_{0}+B_{1}{\text{Education}}_{i}+B_{2}{\text{Employment}}_{i}+\\ \;B_{3}{\text{Prior Care}}_{i}+B_{4}(\text{Facility}\;{\text{Type}}_{i}+\\ \;B_{5}\;{\text{Insurance Status}}_{i}\;\varepsilon \end{array}$

Where:

( $E[\text{cost}\;{\text{of illness}}_{i}]$ ) = Expected cost of illness

Ɛ = Error term including overdispersion

$B_{0}$ = Constant term

$B_{1}B_{2}B_{3}B_{4}B_{5}$ = regression coefficients expressed as incidence rate ratios, which reflect proportional changes in cost due to a unit change in the independent variables

The outputs are presented using tables and graphs. The cost was presented in US dollars using a conversion rate of 129.24, derived from the Central Bank of Kenya as of September 2025.

## 3 Results

### 3.1 Variables

Table 2 presents descriptive statistics of socioeconomic and health-seeking variables from a sample of 100 caregivers for children under 5. The majority of the caregivers who determined healthcare-seeking decisions had attained a secondary level of education, making up 61% of the caregivers, while those with a college/tertiary level of education made up 12% of the caregivers. Caregivers with primary school education levels made up 26%, and those without any level of education made up 1%.

**Table 2:** Variables.

Variables	*N*/100
Education level	
College/Tertiary level	12
Secondary level	61
Primary school level	26
Not gone to school	1
Employment status	
Formally employed	12
Informal employment (Casual laborers, self-employed)	23
Unemployed	65
Prior care seeking	
Direct to Mukuru facilities	53
Community health worker	2
Health facility/ outpatient	8
Others	1
Pharmacist	36
Type of health facility visited	
Lady of Nazareth Mukuru Njenga	5
MCC Mukuru Health Centre	32
Medical Missionaries of Mary	42
NMS Mukuru Reuben Health Centre	21
Insurance status	
Insured	34
Non-Insured	66

Caregivers’ level of employment was categorized as formally employed, those in professions or employed in businesses with regular wages, who made up 12% of the caregivers. Informally employed were 23%, and they consisted of casual laborers and self-employed caregivers. The unemployed caregivers made up 65%, including housewives.

53% of caregivers did not seek prior care elsewhere and presented themselves directly to Mukuru Health facilities. Of the 47% that sought prior care, 36% visited the pharmacy, 8% visited other health facilities, 2% visited community health workers, and a small proportion (1%) sought care from other sources, such as herbalists.

Four different facilities were mapped out for this study: 42% of the caregivers sought care from a private hospital, Medical Missionaries of Mary, while 58% sought care at public facilities, with the majority preferring the MCC Mukuru Health Centre at 32%, NMS Mukuru Reuben Health Centre at 21%, and Lady of Nazareth Mukuru Njenga Hospital at 5%.

The majority of the caregivers (66%) were not insured by NHIF/SHA compared to the insured (34%).

### 3.2 Overall direct and indirect cost of rotavirus diarrhea illness in Mukuru

**Figure 2:**
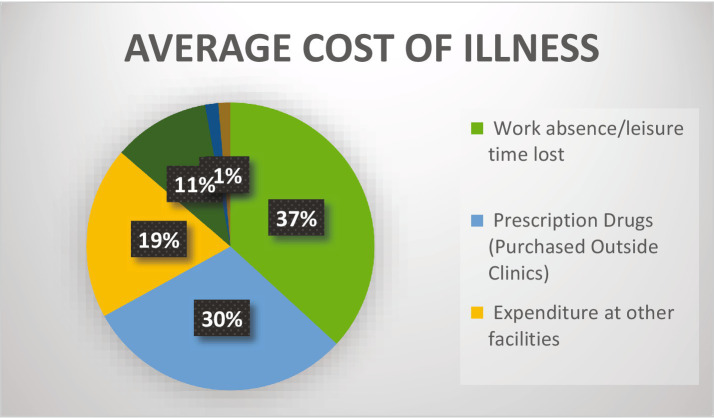
Overall average cost of illness.

A descriptive analysis was conducted to determine the overall cost of illness, categorized into direct medical costs, direct non-medical costs, and indirect costs (Figure 2). The overall expenditure was further compared between the insured and non-insured, as shown in Table 3.

**Table 3:** Overall cost of illness among insured and non-insured caregivers.

Cost category	Insured *n* = 34				Non-insured *n* = 66				Overall cost of illness			
	Mean 95% CI	SD	Median	IQR	Mean 95% CI	SD	Median	IQR	Mean 95% CI	SD	Median	IQR
Direct medical cost												
Expenditure at other facilities	3.26 (0.00, 7.90)	13.29	0.00	0.77	0.92 (0.46, 1.39)	1.90	0.00	0.93	1.72 (0.15, 3.29)	7.91	0.00	0.93
Expenditure at the clinic (drugs, registration)	0.96 (0.30, 1.61)	1.88	0.00	1.16	0.96 (0.61, 1.31)	1.40	0.00	1.93	0.96 (0.65, 1.27)	1.57	0.00	1.74
Prescription drugs (Purchased Outside Clinics)	2.89 (2.13, 3.66)	2.20	2.73	3.95	2.57 (2.03, 3.10)	2.18	2.40	3.02	2.68 (2.24, 3.11)	2.18	2.48	3.39
Direct non-medical cost								0.00				
Transportation	0.16 (0.05, 0.28)	0.34	0.00	0.00	0.11 (0.00, 0.31)	0.77	0.00	0.15	0.13 (0.00, 0.26)	0.66	0.00	0.00
Other costs (meals, airtime, etc.)	0.15(0.04, 0.26)	0.31	0.00	0.15	0.10 (0.06, 0.15)	0.20	0.00	1.67	0.12 (0.07, 0.17)	0.24	0.00	0.15
Indirect costs												
Work absence/leisure time lost	3.49 (2.82, 4.16)	1.93	3.44	1.37	3.15 (2.82, 3.49)	1.36	3.13	3.99	3.27 (2.95, 3.58)	1.58	3.31	1.63
Total	10.92 (6.45, 15.38)	12.80	7.96	6.45	7.83 (6.83, 8.82)	4.04	6.53	3.90	8.88 (7.25, 10.51)	8.22	6.90	4.69

The average cost of treating an episode of diarrhea was $8.88 (95% CI: 7.25, 10.51). This cost comprised direct medical costs, direct non-medical costs, and indirect costs. Direct medical costs accounted for 60.36% of the total cost, with an aggregate mean of $5.36. Expenditure that patients incurred at other facilities before presenting at the Mukuru facilities cost an average of $1.72(95% CI: 0.15, 3.29), which was 19.37% of the total cost. The cost at the Mukuru facilities for drugs and registration was $0.96 (95% CI: 0.65, 1.27), accounting for 10.81% of the total costs. Drugs prescribed for purchase outside the clinics accounted for 30.18% of the total cost, with an average cost of $2.68 (95% CI: 2.25, 3.11). The direct non-medical cost was significantly low, at 2.82% of the total cost, including transportation and incidentals. The indirect costs accounted for 36.82%, with an average income lost due to health-seeking at $3.27 (95% CI: 2.95, 3.58) per episode.

**Figure 3:**
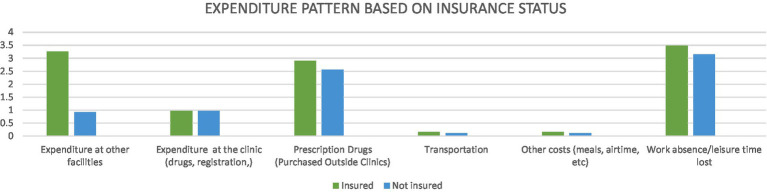
Expenditure patterns based on insurance status.

The insured represented 34% of the caregivers, and they incurred the highest out-of-pocket costs, accounting for 58% of the overall cost of illness. The uninsured comprised 59% of the caregivers and accounted for 42% of the overall cost. Both groups exhibited almost similar patterns of expenditure across all direct and indirect costs, including registration and prescription costs (Figure 3). The major difference in expenditure was on prior care seeking, where the insured spent more at an average cost of $3.26 (95% CI: 0.00, 7.90) compared to the uninsured, who spent $0.92 (95% CI: 0.46, 1.39).

### 3.3 Expenditure patterns based on socioeconomic status and health-seeking practices

A descriptive analysis on how the cost of illness varies based on health-seeking practices and socioeconomic status was carried out. The costs were stratified based on education level, employment status, prior care-seeking status, and the types of hospital facilities that were visited.

#### 3.3.1 Expenditure patterns based on education level

**Figure 4:**
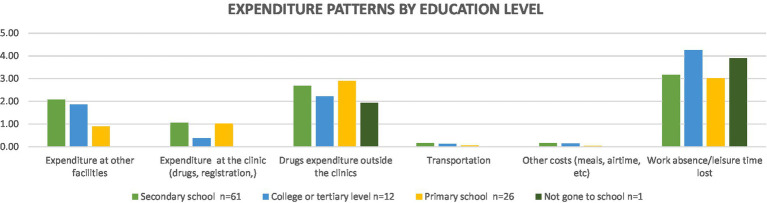
Expenditure patterns by education level.

Caregivers with college/tertiary and secondary levels of education registered the highest expenditure with a mean of $9.01(95% CI: 5.94, 12.07) and $9.30 (95% CI: 6.76, 11.85), respectively (Supplementary Table S1). Across all education levels, costs were primarily driven by purchases of drugs outside health facilities and opportunity costs (Figure 4). Primary-educated caregivers incurred moderately lower total costs, $7.93 (95% CI: 6.20, 9.66), but spent the most on prescription drugs overall, with comparable expenditures between prior care seeking and care at Mukuru facilities. Those with no formal education incurred the lowest overall cost of $5.83.

#### 3.3.2 Expenditure patterns based on employment status

**Figure 5:**
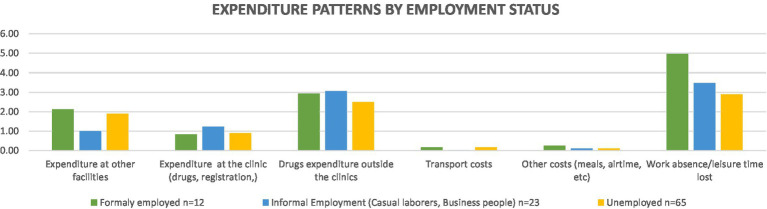
Expenditure patterns by employment status.

The formally employed caregivers spent the highest, with a mean of $11.27 (95% CI: 8.51, 14.03). 74, largely driven by indirect costs and the purchase of drugs outside the clinic (Supplementary Table S2). They also reported the highest direct costs in prior care seeking at $2.13 (95% CI: 0.48, 2.67) compared to costs at the Mukuru facilities, $0.84 (95% CI: 0.35, 1.32). Informally employed caregivers followed with a mean cost of approximately $8.87(95% CI: 7.26, 10.48), with the highest spending on out-of-facility drug purchases and relatively higher consultation and registration costs at Mukuru facilities. Unemployed caregivers incurred the lowest overall costs, with a mean of $8.44 (95% CI: 6.02, 10.85), and their expenditure was mainly driven by purchases of drugs outside clinics and moderate spending on prior care seeking, with minimal costs incurred at the study facilities. Across all employment categories, productivity losses and drug purchases outside facilities were the primary drivers of total household expenditure, with productivity losses being the highest among the formally employed caregivers (Figure 5).

#### 3.3.3 Expenditure patterns based on prior care-seeking behavior

**Figure 6:**
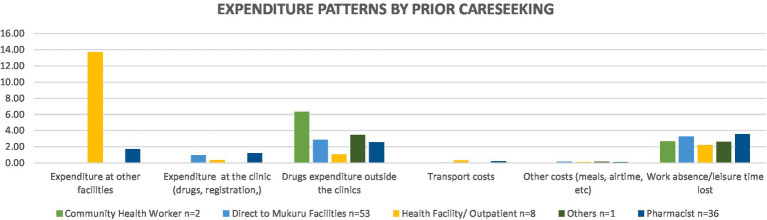
Expenditure patterns by prior care seeking.

Caregivers who sought care directly at Mukuru clinic spent $7.26 (95% CI: 6.29, 8.23), largely driven by income loss and drug purchases outside the clinics (Supplementary Table S3). Those who first sought care at other health facilities and later presented at Mukuru clinics incurred the highest mean costs, $17.72 (95% CI: 0.00, 39.00), despite minimal expenditure at the Mukuru facilities. Caregivers who visited pharmacies accounted for $9.36 (95% CI: 7.844, 10.89) and recorded the highest income loss and substantially higher total drug expenditures due to self-medication and clinic prescriptions. Those who consulted community health workers incurred moderate total costs, $8.95 (95%: 8.66, 9.24), driven almost entirely by drug purchases and indirect costs. Overall, income loss and drug purchases outside formal health facilities were the main drivers of expenditure, particularly among caregivers who were seeking care from multiple providers (Figure 6).

#### 3.3.4 Expenditure patterns based on health facilities visited

**Figure 7:**
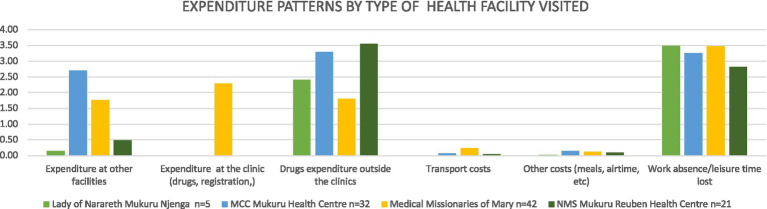
Expenditure patterns by type of health facilities visited.

Clear cost differences were evident between private and public facilities (Supplementary Table S4). The private hospital, Medical Missionaries of Mary, attracted the largest share of caregivers and recorded the highest mean cost of $9.70 (95%: 8.18, 11.21), driven mainly by registration and consultation fees, despite slightly higher transport costs and the lowest spending on drugs outside the facility. In contrast, public facilities showed more variable but generally lower costs, with MCC Mukuru Health Centre recording the highest mean cost among public facilities of $9.48 (95% CI: 4.73, 14.22), largely due to costs incurred before presenting at the study clinics, while Lady of Nazareth Mukuru Njenga Hospital and NMS Mukuru Reuben Health Centre recorded lower mean costs, $6.08 (95%:2.15, 10.00) and $6.99 (95% CI: 5.73, 8.25), respectively. Although public facilities charged no fees for registration or drugs, caregivers incurred higher expenditures on drug purchases outside clinics, reflecting limited drug availability. Across all facilities, indirect costs were substantial, with the highest productivity losses observed among caregivers attending the Lady of Nazareth Mukuru Njenga Hospital (Figure 7).

### 3.4 Key drivers of the cost of illness

The study also aimed at testing how education level, employment status, types of health facilities visited, and prior care-seeking behavior drive the total cost of rotavirus diarrhea using a gamma log link model. Goodness-of-fit statistics were calculated to assess the adequacy of the model (Table 4).

**Table 4:** Goodness of fit tests.

Description	Value
Number of observations	100
Residual degrees of freedom	88
Scale parameter	0.37
Deviance	25.19
Deviance/df	0.29
Pearson chi-square	31.88
Pearson/df	0.36
Log pseudolikelihood	−313.35
Akaike Information Criterion (AIC)	6.51
Bayesian Information Criterion (BIC)	−380.07

The deviance statistic was 25.19 with a deviance-to-degree-of-freedom ratio of 0.29, and the Pearson chi-square statistic was 31.88 with a Pearson degree of freedom ratio of 0.36. The low ratios (below 1) indicated that the Gamma model adequately captured the variability and heteroscedastic structure of the cost data. The model indicated a moderate level of unexplained variability, with a scale parameter of 0.37, which is consistent with the expected heteroskedasticity of the cost data. The goodness-of-fit statistics demonstrated that the gamma log link function provided an appropriate framework for modeling the cost data. The coefficients from the gamma log link were interpreted as cost ratios, representing the expected change in cost due to a unit change in the predictor variables, holding all other variables constant. Table 5 presents the regression outputs on how socioeconomic characteristics and health-seeking patterns influenced the cost of rotavirus diarrheal illness.

**Table 5:** Cost drivers of diarrheal illnesses.

Mean cost	Exp (b) Cost ratio (CR)	Std. Err.	*z*	*p* > z	[95% Conf. interval]
Type of health facility visited					
Medical Missionaries of Mary (private)	Ref				
Lady of Nazareth Mukuru Njenga	0.73	0.23	−1.00	0.32	0.39–1.36
MCC Mukuru Health Centre	0.91	0.12	−0.73	0.47	0.70–1.17
NMS Mukuru Reuben Health Centre	0.73	0.09	−2.54	0.01	0.57–0.93
Employment status					
Formally employed	Ref				
Informally employed	0.99	0.18	−0.05	0.96	0.69–1.41
Unemployed	0.82	0.15	−1.05	0.29	0.57–1.19
Education status					
Tertiary/college level	Ref				
Not gone to school	1.25	0.42	0.66	0.51	0.65–2.42
Primary school	1.10	0.20	0.55	0.58	0.78–1.56
Secondary level	1.18	0.21	0.98	0.33	0.84–1.66
Prior care seeking					
Direct to Mukuru health facilities	Ref				
Community health worker	1.58	0.20	3.65	0.00	1.24–2.02
Health facility/outpatient	2.26	1.06	1.74	0.08	0.90–5.65
Others	0.90	0.11	−0.84	0.40	0.71–1.14
Pharmacist	1.22	0.12	2.02	0.04	1.01–1.49
Insurance status					
Insured	Ref				
Not insured	0.88	0.10	−1.13	0.26	0.70–1.10

Based on the types of health facilities visited, respondents who sought care at the NMS Mukuru Reuben Health Centre showed a statistically significant difference in costs compared to the reference private facility (*p* = 0.05, 95% CI: 0.57–0.93). They had a cost ratio of 0.73 and 27% lower costs than the Medical Missionaries of Mary, a private facility. Visiting the Lady of Nazareth Mukuru Njenga Hospital (*p* = 0.32, 95% CI: 0.39–1.36) and MCC Mukuru Health Centre CR 0.91 (*p* = 0.47, 95% CI: 0.70–1.17) showed no statistically significant costs compared to the private facility. However, visiting the Lady of Nazareth Mukuru Njenga Hospital (CR 0.73) lowered costs by 27% compared to visiting a private facility. However, the lack of statistical significance indicates that the difference could have been due to chance.

Based on employment status, being informally employed (CR 0.99 *p* = 0.96: 95% CI 0.69–1.41) was associated with a 1% reduction in expenditure compared to employed caregivers. Being unemployed (CR 0.82, *p* = 0.29: 95% CI 0.57–1.19) was associated with an 18% reduction in expenditure. However, they did not show any significant difference compared with the employed reference group.

All the categories in the education status did not demonstrate any significant difference in cost compared to those with tertiary/college qualifications: Secondary level (CR:1.18, *p* = 0.33, 95% CI: 0.84–1.66), primary level (CR 1.10, *p* = 0.58 95% CI: 0.78–1.561), not gone to school (CR = 1.25, *p* = 0.66: 95% CI: 0.65–2.42). They were associated with an 18, 10, and 25% increase in expenditure, respectively, compared to the college level, but only by chance.

Comparing the first point of contact before presenting at the Mukuru facilities, those who visited community health workers (CR = 1.58, *p* = 0.00, 95% CI: 1.24–2.02) and pharmacists (CR = 1.22, *p* = 0.04, 95% CI: 1.01–1.49) registered statistically significant cost differences compared to those who presented directly to Mukuru health facilities with an increased rate of 58 and 22%, respectively.

Visiting other health facilities as a first point of contact did not show statistically significant cost differences, but patients spent more than twice the cost of those who presented directly to the health facilities in Mukuru (CR = 2.26, *p* = 0.00, 95% CI: 1.62–3.45).

Visiting other sources such as herbalists (CR = 0.90, *p* = 0.40, 95% CI: 0.71–1.14) had no significant association with costs compared to first presenting at the Mukuru facilities.

Non-insured caregivers (CR = 0.88, *p* = 0.26, 95% CI: 0.70–1.10) spent more time than insured caregivers by 12%, although the difference was not statistically significant.

## 4 Discussion

The average cost of treating a diarrheal episode was estimated at $8.88(95% CI: 7.25, 10.51) per diarrheal episode. The cost represents a substantial economic burden for households, especially for vulnerable urban informal settlement residents who earn an average monthly income of $93–$100 per month (54, 55). Therefore, they spent approximately 8.9%–9.5% of their monthly income on treating rotavirus diarrhea illnesses. This highlights the substantial economic burden of childhood diarrhea in low-income urban settings and aligns with evidence from other low-income urban settings in LMICs, where households spend between 4 and 17% of their monthly income per diarrheal episode (22, 56).

Direct medical costs accounted for the largest share of expenditures, driven primarily by purchases of drugs outside health facilities and costs incurred at other facilities prior to presentation at Mukuru clinics. Although only a small proportion of total costs were incurred within Mukuru facilities, limited drug availability shifted a significant economic burden to households’ purchases, reflecting health system inefficiencies and fragmented service delivery.

Indirect costs related to income loss constituted over one-third of total expenditure, highlighting the economic impact of caregiving time in a predominantly low-income population and consistent with human capital theory, which conceptualizes health as an asset influencing productivity (38, 57, 58).

Insurance coverage did not translate into lower household expenditures. Insured caregivers incurred slightly higher costs, particularly those related to prior care seeking, reflecting greater readiness to seek formal healthcare. However, persistent drug stockouts in facilities continue to expose insured households to out-of-pocket spending, limiting the financial protection role of insurance. Uninsured caregivers appeared more likely to delay care, potentially lowering reported costs but raising concerns about delayed treatment and adverse health outcomes. The findings were consistent with Andersen’s healthcare utilization framework, which stipulates that enabling resources such as insurance may not automatically translate to reduced economic burden when health system constraints persist (59).

Education and employment status shaped expenditure patterns descriptively but were not statistically significant cost drivers. Caregivers with secondary and tertiary education incurred higher costs, largely driven by indirect costs and the use of multiple care pathways, reflecting both higher opportunity costs and greater willingness to invest in healthcare. Conversely, caregivers with no formal education incurred the lowest costs, likely reflecting their constrained ability to pay rather than a lower need. Similarly, employed caregivers faced higher costs due to income loss and diversified care seeking, whereas unemployed caregivers exhibited skewed spending, with a subset incurring disproportionately high costs despite limited resources. Those with higher education, by seeking informal care that increased overall costs, were able to make treatment choices that were more effective and reduced illness severity. Hence, when they finally sought care at Mukuru clinics, the illness did not cost them much. These findings suggest shared socioeconomic vulnerability across groups in under-resourced area settings. The findings also align with evidence that socioeconomic inequalities shape both health-seeking behavior and financial burden in urban informal settlements (23, 60).

Prior care seeking at pharmacies and community health workers emerged as major drivers of cost. Caregivers who sought care at other health facilities before presenting at Mukuru incurred more than twice the cost of those who presented directly, highlighting the financial consequences of delayed and sequential care seeking. However, despite the higher overall expenditure, these caregivers incurred substantially lower costs once they presented at Mukuru clinics, suggesting that initial interventions at formal health facilities may have reduced illness severity and the need for intensive or costly care at study facilities. However, the lack of any statistical significance indicates that the difference could have been due to chance. Visiting pharmacies first was also associated with higher costs, driven by self-medication and subsequent facility visits, while care through community health workers was linked to increased costs but lower productivity losses, suggesting a role in early detection rather than treatment.

Clear differences were observed between public and private facilities. The private hospital attracted the largest share of caregivers and registered the highest costs, largely due to consultation and registration fees; however, it was associated with lower out-of-facility drug purchases, indicating better drug availability. Visiting public facilities registered lower costs compared to visiting private facilities. However, despite shielding caregivers from user fees, the facilities exposed households to higher external drug costs due to stockouts.

These cost estimates and patterns observed in this study demonstrated that expenditure on diarrheal care is influenced not only by illness severity but also by health-seeking behavior, facility type, and socioeconomic status, reflecting inequities in access and financial protection (22, 32, 56).

## 5 Study limitations

1. The main limitation of this study is the focus on outpatient costs to determine the out-of-pocket costs incurred by households, excluding inpatient costs, which may not provide a robust picture of the cost of diarrhea.
2. Data were collected at the clinics in Mukuru, which only captured costs incurred at the clinic during the treatment of diarrhea and drug costs but did not include follow-up costs such as continued productivity loss after the clinic or home-based expenditures. A follow-up household survey would ensure that these additional costs are captured.
3. Variables such as disease severity and age were not captured, and given their possibility to influence care seeking and subsequent costs, their omission may have introduced confounding.
4. Indirect costs were estimated using minimum wages in line with human capital theory. This approach was applied to both employed and non-employed individuals, which could have underestimated the productivity loss for employed caregivers.

## 6 Conclusion

Overall, the findings indicate that the cost of childhood diarrhea in Mukuru is driven less by caregivers’ socioeconomic characteristics and more by health system factors, particularly drug availability and fragmented care pathways. Strengthening drug supply chains, improving the quality and reliability of public primary healthcare, and promoting timely and proper care-seeking pathways could substantially reduce household costs and improve financial protection in urban informal settlements.

While the introduction of the rotavirus vaccination policy was a critical initiative aimed at reducing the burden, the study underscored the importance of integrating other socioeconomic and health system strategies and policies towards the control of both the clinical and economic burden of diarrhea.

## 7 Recommendations

- While the Social Health Authority and Primary Healthcare Fund have expanded access to free primary healthcare, the lack of drug availability continues to shift treatment costs to households that have to purchase drugs out of pocket. The findings suggest the need to strengthen drug supply chains in primary care facilities serving informal settlements to ensure that essential medicines are consistently available and to reduce out-of-pocket expenses for households.
- Promote timely and efficient care seeking through targeted health education programs that guide caregivers on proper pathways, reducing delays, inefficiencies, and productivity losses associated with fragmented care.
- Incorporate economic and equity indicators into policies and surveillance by systematically tracking household costs, income levels, and employment status alongside clinical data. This will help identify the populations most financially affected and inform equitable, evidence-based interventions to reduce both the clinical and economic burden of diarrhea.
- Integrate informal healthcare providers into primary care networks by linking pharmacies, community health promoters, and other informal centers with public clinics. Equip community health workers with ORS and basic kits to enable timely and coordinated responses, reduce unnecessary referrals, and lower household costs.
- Conduct comprehensive studies on inpatient diarrhea costs to capture the full economic impact of severe cases, enabling policymakers to design interventions that address both outpatient and hospitalization expenses.

## Data Availability

The raw data supporting the conclusions of this article will be made available by the authors without undue reservation.

## References

[1] World Health Organization (2024). World health statistics 2024: monitoring health for the SDGs, sustainable development goals. Available online at: https://www.who.int/publications/i/item/9789240094703 (Accessed February 3, 2026)

[2] Kyu HH Vongpradith A Dominguez R-MV Ma J Albertson SB Novotney A . Global, regional, and national age-sex-specific burden of diarrhoeal diseases, their risk factors, and aetiologies, 1990–2021, for 204 countries and territories: a systematic analysis for the global burden of disease study 2021. Lancet Infect Dis. (2025) 25:519–36. doi: 10.1016/S1473-3099(24)00691-1, 39708822 PMC12018300

[3] Mulatya DM Ochieng C. Disease burden and risk factors of diarrhoea in children under five years: evidence from Kenya’s demographic health survey 2014. Int J Infect Dis. (2020) 93:359–66. doi: 10.1016/j.ijid.2020.02.003, 32061860

[4] Victor N Gerryshom M Njuguna M. Assessing household vulnerability to climate risks in Mukuru special planning area (SPA), Nairobi: a mixed-methods spatial analysis. Afr Habitat Rev. (2025) 20:3580–97. Available online at: http://uonjournals.uonbi.ac.ke/ojs/index.php/ahr

[5] Sunikka-Blank M Bardhan R Musya M Njoroge P Nyaga M Weru J. Cultural dimensions of energy transitions and clean cooking in informal settlements: the case of Mukuru, Nairobi. Soc Change. (2026) 56:111–29. doi: 10.1177/00490857251413422

[6] Kiiru S Maina J Mwaniki JN Songoro E Kariuki S. Enteric bacterial agents associated with diarrhea and their antimicrobial sensitivity profiles in children under 5 years from Mukuru informal settlement, Nairobi, Kenya. BMC Infect Dis. (2024) 24:237. doi: 10.1186/s12879-024-09114-5, 38388369 PMC10882725

[7] Osano B Kamenwa R Wamalwa D Wang’ombe J. Short term clinical outcome of children with rotavirus infection at Kenyatta National Hospital, Nairobi. East Afr Med J. (2011) 87:242–7. doi: 10.4314/eamj.v87i6.63082, 23057266

[8] Wandera EA Mohammad S Bundi M Komoto S Nyangao J Kathiiko C . Impact of rotavirus vaccination on rotavirus and all-cause gastroenteritis in peri-urban Kenyan children. Vaccine. (2017) 35:5217–23. doi: 10.1016/j.vaccine.2017.07.096 28780116

[9] Muendo C Laving A Kumar R Osano B Egondi T Njuguna P. Prevalence of rotavirus infection among children with acute diarrhoea after rotavirus vaccine introduction in Kenya, a hospital cross-sectional study. BMC Pediatr. (2018) 18:323. doi: 10.1186/s12887-018-1291-8, 30309343 PMC6180366

[10] Sifuna P Shaw AV Lucas T Ogutu B Otieno W Larsen DA. Deployment of rotavirus vaccine in Western Kenya coincides with a reduction in all-cause child mortality: a retrospective cohort study. Vaccine. (2023) 11:1299. doi: 10.3390/vaccines11081299, 37631867 PMC10458991

[11] Otieno GP Bottomley C Khagayi S Adetifa I Ngama M Omore R . Impact of the introduction of rotavirus vaccine on hospital admissions for diarrhea among children in Kenya: a controlled interrupted time-series analysis. Clin Infect Dis. (2020) 70:2306–13. doi: 10.1093/cid/ciz912, 31544211 PMC7245159

[12] Ministry of Health Kenya. Policy Guidelines on Control and Management of Diarrhoeal Diseases in Children Below Five Years in kenya. (2104). Available online at: http://guidelines.health.go.ke/#/category/13/26/meta (Accessed May 11, 2026).

[13] Ministry of Health Kenya. Immunization in Kenya: A guide for Health Workers. (2010). Available online at: http://guidelines.health.go.ke/#/category/13/7/meta (Accessed May 11, 2026).

[14] Kenya National Bureau of Statistics. Kenya Demographic and Health Survey - 2022. (2023). Available online at: https://www.knbs.or.ke/reports/kdhs-2022/ (Accessed May 11, 2026).

[15] Mberu B Simiyu S Gutema FD Sewell D Busienei PJ Tumwebaze IK . Landscape analysis of the Kenyan policy on the treatment and prevention of diarrheal disease among under-5 children. BMJ Open. (2024) 14:e081906. doi: 10.1136/bmjopen-2023-081906 PMC11337661 39160109

[16] WHO Immunization Data portal - African Region. Immun data. Available online at: https://immunizationdata.who.int/dashboard/regions/african-region (Accessed February 26, 2026)

[17] Su TT Flessa S. Determinants of household direct and indirect costs: an insight for health-seeking behaviour in Burkina Faso. Eur J Health Econ. (2013) 14:75–84. doi: 10.1007/s10198-011-0354-7, 21953320

[18] Phiri M Osman R Weldegebriel S Sabola S Ongadi B Beavis C . Exploration of the social determinants of diarrhoea, rotavirus vaccine uptake, and vaccine ‘fatigue’ in Ethiopia, Kenya, and Malawi. PLoS One. (2025) 20:e0319691. doi: 10.1101/2025.02.07.25321869 40924732 PMC12419581

[19] Li Y Chen Z. Health-seeking behavior and patient welfare: evidence from China. China Econ Rev. (2023) 80:102015. doi: 10.1016/j.chieco.2023.102015

[20] Chuma J Gilson L Molyneux C. Treatment-seeking behaviour, cost burdens and coping strategies among rural and urban households in coastal Kenya: an equity analysis. Trop Med Int Health. (2007) 12:673–86. doi: 10.1111/j.1365-3156.2007.01825.x, 17445135

[21] Otieno PO Wambiya EOA Mohamed SM Mutua MK Kibe PM Mwangi B . Access to primary healthcare services and associated factors in urban slums in Nairobi-Kenya. BMC Public Health. (2020) 20:981. doi: 10.1186/s12889-020-09106-5, 32571277 PMC7310125

[22] Buigut S Ettarh R Amendah DD. Catastrophic health expenditure and its determinants in Kenya slum communities. Int J Equity Health. (2015) 14:46. doi: 10.1186/s12939-015-0168-9, 25971679 PMC4438568

[23] Marew A Jebero Zaza Z Birhanu S Belachew A Kasse T. Delays in health care seeking for diarrheal disease and associated factors among caregivers of under five children in health centers of Northwest Ethiopia: a mixed-method study. BMC Public Health. (2025) 25:138. doi: 10.1186/s12889-025-21300-x, 39806357 PMC11731159

[24] Tate JE Rheingans RD O’Reilly CE Obonyo B Burton DC Tornheim JA . Rotavirus disease burden and impact and cost-effectiveness of a rotavirus vaccination program in Kenya. J Infect Dis. (2009) 200:S76–84. doi: 10.1086/605058, 19817618

[25] De Broucker G Sim SY Brenzel L Gross M Patenaude B Constenla DO. Cost of nine pediatric infectious illnesses in low- and middle-income countries: a systematic review of cost-of-illness studies. Pharmaco Economics. (2020) 38:1071–94. doi: 10.1007/s40273-020-00940-4, 32748334 PMC7578143

[26] Ngabo F Mvundura M Gazley L Gatera M Rugambwa C Kayonga E . The economic burden attributable to a child’s inpatient admission for diarrheal disease in Rwanda. PLoS One. (2016) 11:e0149805. doi: 10.1371/journal.pone.0149805, 26901113 PMC4764684

[27] Baral R Nonvignon J Debellut F Agyemang SA Clark A Pecenka C. Cost of illness for childhood diarrhea in low- and middle-income countries: a systematic review of evidence and modelled estimates. BMC Public Health. (2020) 20:619. doi: 10.1186/s12889-020-08595-8, 32370763 PMC7201538

[28] Agoti CN Curran MD Murunga N Ngari M Muthumbi E Lambisia AW . Differences in epidemiology of enteropathogens in children pre- and post-rotavirus vaccine introduction in Kilifi, coastal Kenya. Gut Pathog. (2022) 14:32. doi: 10.1186/s13099-022-00506-z, 35915480 PMC9340678

[29] Shah M Odoyo E Wandera E Kathiiko C Bundi M Miringu G . Burden of rotavirus and enteric bacterial pathogens among children under 5 years of age hospitalized with diarrhea in suburban and rural areas in Kenya. Jpn J Infect Dis. (2017) 70:442–7. doi: 10.7883/yoken.JJID.2016.398, 28250260

[30] Mukiira C Ibisomi L. Health care seeking practices of caregivers of children under 5 with diarrhea in two informal settlements in Nairobi, Kenya. J Child Health Care. (2015) 19:254–64. doi: 10.1177/1367493513508231, 24270995

[31] At Thobari J Sutarman Mulyadi AWE Watts E Carvalho N Debellut F . Direct and indirect costs of acute diarrhea in children under five years of age in Indonesia: health facilities and community survey. Lancet Reg Health - West Pac. (2022) 19:100333. doi: 10.1016/j.lanwpc.2021.100333 35024664 PMC8669319

[32] Rheingans R Kukla M Adegbola RA Saha D Omore R Breiman RF . Exploring household economic impacts of childhood diarrheal illnesses in 3 African settings. Clin Infect Dis. (2012) 55:S317–26. doi: 10.1093/cid/cis763, 23169944 PMC3502313

[33] Gulis G Mulumba JAA Juma O Kakosova B. Health status of people of slums in Nairobi, Kenya. Environ Res. (2004) 96:219–27. doi: 10.1016/j.envres.2004.01.016, 15325882

[34] Muoki M Tumuti D Rombo D. Nutrition and public hygiene among children under five years of age in Mukuru slums of Makadara division, Nairobi. East Afr Med J. (2008) 85:386–97. doi: 10.4314/eamj.v85i8.9656, 19115556

[35] Pourhoseingholi MA Vahedi M Rahimzadeh M. Sample size calculation in medical studies. Gastroenterol Hepatol Bed Bench. (2013) 6:14–7. doi: 10.1159/000322830 24834239 PMC4017493

[36] Martínez-Mesa J González-Chica DA Bastos JL Bonamigo RR Duquia RP. Sample size: how many participants do I need in my research? An Bras Dermatol. (2014) 89:609–15. doi: 10.1590/abd1806-4841.20143705, 25054748 PMC4148275

[37] Zrineh A Al-Usta M Alwawi A. Sampling methods and sample size determination in clinical research: an educational review. J Gen Fam Med. (2026) 27:e70096. doi: 10.1002/jgf2.70096, 41696722 PMC12897549

[38] Bleakley H. Health, human capital, and development. Annu Rev Econ. (2010) 2:283–310. doi: 10.1146/annurev.economics.102308.124436, 24147187 PMC3800109

[39] Drummond MF Sculpher M Torrance GW O’Brien BJ Abe SK Stoddart GL . (2023). Methods for the economic Evaluation of Health care Programmes. 3rd ed. Oxford: Oxford University Press (2007). 379. doi: 10.1093/oso/9780198529446.001.0001

[40] Elliott R Payne K. Essentials of Economic Evaluation in Healthcare. London, UK: Pharmaceutical Press (2005). p. 260.

[41] Johannesson M. Theory and Methods of Economic Evaluation of Health Care. Dordrecht, The Netherlands: Springer Science & Business Media (1996). p. 270.

[42] Pike J Grosse SD. Friction cost estimates of productivity costs in cost-of-illness studies in comparison with human capital estimates: a review. Appl Health Econ Health Policy. (2018) 16:765–78. doi: 10.1007/s40258-018-0416-4, 30094591 PMC6467569

[43] Zhang W Bansback N Anis AH. Measuring and valuing productivity loss due to poor health: a critical review. Soc Sci Med. (2011) 72:185–92. doi: 10.1016/j.socscimed.2010.10.026, 21146909

[44] Choi H-J Lee E-W. "Methodology of estimating socioeconomic burden of disease using National Health Insurance (NHI) data". In: Reddy S Tavares IA, editors. Evaluation of Health Services. London, UK: IntechOpen (2020)

[45] Van Den Hout WB. The value of productivity: human-capital versus friction-cost method. Ann Rheum Dis. (2010) 69:i89–91. doi: 10.1136/ard.2009.117150, 19995754

[46] Setiawan E Cassidy-Seyoum SA Thriemer K Carvalho N Devine A. A systematic review of methods for estimating productivity losses due to illness or caregiving in low- and middle-income countries. PharmacoEconomics. (2024) 42:865–77. doi: 10.1007/s40273-024-01402-x, 38874846 PMC11249595

[47] Sachs J. Macroeconomics and Health: Investing in Health for Economic Development; Report of the Commission on Macroeconomics and Health. Geneva: World Health Organization (2001). p. 200.

[48] Salinas Ruíz J Montesinos López OA Hernández Ramírez G Crossa HJ. "Generalized linear models". In: Salinas Ruíz J Montesinos López OA Hernández Ramírez G Crossa Hiriart J, editors. Generalized Linear Mixed Models with Applications in Agriculture and Biology. Cham: Springer International Publishing (2023). p. 43–84.

[49] Lwin KS Nomura S Yoneoka D Ueda P Abe SK Shibuya K. Associations between parental socioeconomic position and health-seeking behaviour for diarrhoea and acute respiratory infection among under-5 children in Myanmar: a cross-sectional study. BMJ Open. (2020) 10:e032039. doi: 10.1136/bmjopen-2019-032039, 32220909 PMC7170571

[50] Petrinco M Barbati G Meylan D Pagano E Gregori D Merletti F . Robust gamma regression models for the analysis of health care cost data. Model Assist Stat Appl. (2012) 7:115–24. doi: 10.3233/MAS-2011-0218, 42048154

[51] Malehi AS Pourmotahari F Angali KA. Statistical models for the analysis of skewed healthcare cost data: a simulation study. Health Econ Rev. (2015) 5:11. doi: 10.1186/s13561-015-0045-7, 26029491 PMC4442782

[52] Manning WG Basu A Mullahy J. Generalized modeling approaches to risk adjustment of skewed outcomes data. J. Health Econ. (2003) 24:465–88. doi: 10.3386/t0293 15811539

[53] Barber J Thompson S. Multiple regression of cost data: use of generalised linear models. J Health Serv Res Policy. (2004) 9:197–204. doi: 10.1258/1355819042250249, 15509405

[54] Soma K Janssen VCJ Ayuya OI Obwanga B. Food systems in informal urban settlements—exploring differences in livelihood welfare factors across Kibera, Nairobi. Sustainability (Basel). (2022) 14:11099. doi: 10.3390/su141711099

[55] Corburn J Njoroge P Weru J Musya M. Urban climate justice, human health, and citizen science in Nairobi’s informal settlements. Urban Sci (Basel). (2022) 6:36. doi: 10.3390/urbansci6020036

[56] Sultana R Luby SP Gurley ES Rimi NA Swarna ST Khan JAM . Cost of illness for severe and non-severe diarrhea borne by households in a low-income urban community of Bangladesh: a cross-sectional study. PLoS Negl Trop Dis. (2021) 15:e0009439. doi: 10.1371/journal.pntd.0009439, 34115764 PMC8221788

[57] Bloom D Canning D. Health as human capital and its impact on economic performance. Geneva Pap Risk Insur Issues Pract. (2003) 28:304–15. doi: 10.1111/1468-0440.00225

[58] Strauss J Thomas D. Health, nutrition, and economic development. J Econ Lit. (1998) 36:766–817.

[59] Alkhawaldeh A ALBashtawy M Rayan A Abdalrahim A Musa A Eshah N . Application and use of Andersen’s behavioral model as theoretical framework: a systematic literature review from 2012–2021. Iran J Public Health. (2023) 52:1346–54. doi: 10.18502/ijph.v52i7.13236, 37593505 PMC10430393

[60] Fikire A Ayele G Haftu D. Determinants of delay in care seeking for diarrheal diseases among mothers/caregivers with under-five children in public health facilities of Arba Minch town, southern Ethiopia; 2019. PLoS One. (2020) 15:e0228558. doi: 10.1371/journal.pone.0228558, 32053615 PMC7018063

