# Supplementary material for: How health systems factors, socioeconomic status, and care-seeking practices influence the cost of rotavirus-related diarrhea care in Mukuru informal settlements, Kenya
Source: Front Public Health. 2026 Jul 24;14:1828318. doi: 10.3389/fpubh.2026.1828318 (PMC13449940; doi:10.3389/fpubh.2026.1828318)
Supplement: Supplementary file 1 [file Table_1.docx]

**SUPPLEMENTARY TABLES**

**Supplementary Table S1: Cost of illness based on education level**

| **COST CATEGORIES** | College or tertiary level n=12 | | | | Secondary school n=61 | | | | Primary school n=25 | | | | Not gone to school n=1 | | | |
| --- | --- | --- | --- | --- | --- | --- | --- | --- | --- | --- | --- | --- | --- | --- | --- | --- |
| **Direct Medical Cost** | Mean 95% CI | SD | Median | IQR | Mean 95% CI | SD | Median | IQR | Mean 95% CI | SD | Median | IQR | Mean 95% CI | SD | Median | IQR |
| Expenditure at other facilities | 1.86 (1.03, 4.13) | 0.00 | 0.00 | 0.00 | 2.07 (0.00, 4.61) | 0.00 | 0.00 | 0.00 | 0.89 (0.11, 1.68) | 1.95 | 0.00 | 0.77 | 0.00 (0.00, 0.00) | 0.00 | 0.00 | 0.00 |
| Expenditure at the clinic (drugs, registration,) | 0.39 (0.00, 0.78) | 0.00 | 0.00 | 0.00 | 1.06 (0.65, 1.48) | 0.00 | 0.00 | 0.00 | 1.03 (0.31, 1.73) | 1.76 | 0.00 | 1.93 | 0.00 (0.00, 0.00) | 0.00 | 0.00 | 0.00 |
| Prescription Drugs (Purchased Outside Clinics) | 2.22 (0.92, 3.52) | 0.00 | 0.00 | 0.00 | 2.69 (2.10, 3.27) | 0.00 | 0.00 | 0.00 | 2.90 (2.08, 3.72) | 2.03 | 3.21 | 3.10 | 1.93 | 0.00 | 1.93 | 0.00 |
| **Direct Non-Medical Cost** |  |  |  |  |  |  |  |  |  |  |  |  |  |  |  |  |
| Transportation | 0.13 (0.00, 0.32) | 0.00 | 0.00 | 0.00 | 0.17 (0.00, 0.38) | 0.00 | 0.00 | 0.00 | 0.06 (0.00, 0.13) | 0.16 | 0.00 | 0.00 | 0.00 (0.00,0.00) | 0.00 | 0.00 | 0.00 |
| Other costs (meals, airtime, etc) | 0.15 (0.00, 0.41) | 0.00 | 0.00 | 0.00 | 0.16 (0.10, 0.22) | 0.00 | 0.00 | 0.00 | 0.03 (0.00, 0.05) | 0.06 | 0.00 | 0.00 | 0.00 (0.00,0.00) | 0.00 | 0.00 | 0.00 |
| **Indirect Costs** |  |  |  |  |  |  |  |  |  |  |  |  |  |  |  |  |
| Work absence/leisure time lost | 4.27 (2.49, 6.04) | 0.00 | 0.00 | 0.00 | 3.17  (2.83, 3.50) | 1.31 | 3.03 | 1.44 | 3.02 (2.49, 3.56) | 1.32 | 3.03 | 1.51 | 3.90 | 0.00 | 3.90 | 0.00 |
| **TOTAL** | **9.01 (5.94, 12.07)** | **4.82** | **9.09** | **6.15** | **9.30 (6.76, 11.85)** | **9.94** | **7.08** | **4.40** | **7.93 (6.20, 9.66)** | **4.29** | **6.71** | **3.62** | **5.83** | **0.00** | **5.83** | **0.00** |
