# Supplementary material for: How health systems factors, socioeconomic status, and care-seeking practices influence the cost of rotavirus-related diarrhea care in Mukuru informal settlements, Kenya
Source: Front Public Health. 2026 Jul 24;14:1828318. doi: 10.3389/fpubh.2026.1828318 (PMC13449940; doi:10.3389/fpubh.2026.1828318)
Supplement: Supplementary file 2 [file Table_2.docx]

**Supplementary Table S2: Cost of illness based on employment status**

| **COST CATEGORIES** | Formally employed n=12 | |  |  | Informally Employed n=23 | |  |  | Unemployed n=65 |  |  |  |
| --- | --- | --- | --- | --- | --- | --- | --- | --- | --- | --- | --- | --- |
| **Direct Medical Cost** | Mean 95% CI | SD | Median | IQR | Mean 95% CI | SD | Median | IQR | Mean 95% CI | SD | Median | IQR |
| Expenditure at other facilities | 2.13 (0.00, 4.34) | 3.48 | 0.62 | 1.93 | 1.01 (0.10, 1.92) | 2.11 | 0.00 | 0.93 | 1.89 (0.00, 4.28) | 9.64 | 0.00 | 0.77 |
| Expenditure at the clinic (drugs, registration,) | 0.84 (0.35, 1.32) | 0.75 | 0.97 | 1.35 | 1.22 (0.38, 2.05) | 1.95 | 0.00 | 1.93 | 0.90 (0.51, 1.28) | 1.54 | 0.00 | 1.55 |
| Prescription Drugs (Purchased Outside Clinics) | 2.93 (0.75, 5.11) | 3.43 | 0.46 | 4.95 | 3.05 (2.03, 4.06) | 2.35 | 2.94 | 3.87 | 2.50 (2.05, 2.95) | 1.83 | 2.48 | 2.71 |
| **Direct Non-Medical Cost** |  |  |  |  |  |  |  |  |  |  |  |  |
| Transportation | 0.16 (0.00, 0.36) | 0.31 | 0.00 | 0.19 | 0.02 (0.00, 0.72) | 0.11 | 0.00 | 0.00 | 0.16 (0.00, 0.36) | 0.80 | 0.00 | 0.00 |
| Other costs (meals, airtime, etc.) | 0.24 (0.00, 0.51) | 0.42 | 0.00 | 0.43 | 0.09 (0.01, 0.20) | 0.25 | 0.00 | 0.15 | 0.11 (0.06, 0.15) | 0.18 | 0.00 | 0.15 |
| **Indirect Costs** |  |  |  |  |  |  |  |  |  |  |  |  |
| Work absence/leisure time lost | 4.97 (3.29, 6.65) | 2.64 | 5.33 | 2.58 | 3.48 (2.85, 4.11) | 1.47 | 3.53 | 2.52 | 2.88 (2.61, 3.15) | 1.09 | 2.60 | 1.49 |
| **Total** | **11.27 (8.51, 14.03)** | 4.34 | 10.74 | 8.03 | **8.87 (7.26, 10.48)** | 3.72 | 7.79 | 5.12 | **8.44 (6.02, 10.85)** | 9.75 | 6.24 | 4.10 |
