# Supplementary material for: How health systems factors, socioeconomic status, and care-seeking practices influence the cost of rotavirus-related diarrhea care in Mukuru informal settlements, Kenya
Source: Front Public Health. 2026 Jul 24;14:1828318. doi: 10.3389/fpubh.2026.1828318 (PMC13449940; doi:10.3389/fpubh.2026.1828318)
Supplement: Supplementary file 3 [file Table_3.docx]

**Supplementary Table S3 :Cost of illness based on care-seeking behaviour**

| **COST CATEGORES** | Direct to Mukuru Clinics n=53 | |  |  | Community Health Workers n=2 | | |  | Other Health Facilities n=8 |  |  |  | Others (herbalist) n=1. | | |  | Pharmacists n=36 | | |  |
| --- | --- | --- | --- | --- | --- | --- | --- | --- | --- | --- | --- | --- | --- | --- | --- | --- | --- | --- | --- | --- |
| **Direct Medical Cost** | Mean 95% CI | SD | Median | IQR | Mean 95% CI | SD | Median | IQR | Mean 95% CI | SD | Median | IQR | Mean 95% CI | SD | Median | IQR | Mean 95% CI | SD | Median | IQR |
| Expenditure at other facilities | 0.00 (0.00,0.00) | 0.00 | 0.00 | 0.00 | 0.00 | 0.00 | 0.00 | 0.00 | 13.72  (0.00, 35.47) | 26.01 | 5.76 | 8.12 | 0.00 | 0.00 | 0.00 | 0.00 | 1.72  (1.06, 2.38) | 1.96 | 1.04 | 1.10 |
| Expenditure at the clinic (drug, registration) | 0.96  (0.49, 1.42) | 1.67 | 0.00 | 1.55 | 0 (0, 0) | 0.00 | 0.00 | 0.00 | 0.34  (0.00, 0.70) | 0.44 | 0.19 | 0.58 | 0.00 | 0.00 | 0.00 | 0.00 | 1.19  (0.65, 1.72) | 1.60 | 0.19 | 1.93 |
| Prescription Drugs (Purchased Outside Clinics) | 2.84  (2.25, 3.44) | 2.17 | 2.79 | 3.02 | 6.31  (0.00, 22.53) | 1.81 | 6.31 | 2.55 | 1.04  (0.07, 2.02) | 1.16 | 0.46 | 1.08 | 3.48 | . | 3.48 | 0.00 | 2.57  (1.83, 3.30) | 2.16 | 2.21 | 3.10 |
| **Direct Non-Medical Cost** |  |  |  |  |  |  |  |  |  |  |  |  |  |  |  |  |  |  |  |  |
| Transport | 0.06  (0.00, 0.12) | 0.22 | 0.00 | 0.00 | 0.00 | 0.00 | 0.00 | 0.00 | 0.29  (0.00, 0.62) | 0.40 | 0.00 | 0.77 | 0.00 | 0.00 | 0.00 | 0.00 | 0.21  (0.00, 0.56) | 1.05 | 0.00 | 0.00 |
| Other costs (meals, airtime, etc.) | 0.13  (0.06, 0.22) | 0.29 | 0.00 | 0.15 | 0.00 | 0.00 | 0.00 | 0.00 | 0.12  (0.00, 0.30) | 0.21 | 0.00 | 0.15 | 0.15 | . | 0.15 | 0.00 | 0.10  (0.05, 0.16) | 0.15 | 0.00 | 0.15 |
| **Indirect Costs** |  |  |  |  |  |  |  |  |  |  |  |  |  |  |  |  |  |  |  |  |
| Work absence/leisure time lost | 3.25  (2.92, 3.59) | 1.21 | 3.53 | 1.37 | 2.65  (0.00,18.58) | 1.77 | 2.65 | 2.51 | 2.21  (0.85, 3.57) | 1.63 | 2.09 | 1.63 | 2.60 | 0.00 | 3.90 | 0.00 | 3.58  (2.91, 4.24) | 1.96 | 3.00 | 1.72 |
| **Total** | **7.26 (6.29, 8.23)** | **3.52** | **6.19** | **3.99** | **8.95 (8.66, 9.24)** | **0.03** | **8.95** | **0.05** | **17.72 (0.00, 39.00)** | **25.48** | **9.52** | **8.42** | **6.24** | **0.00** | **6.24** | **0.00** | **9.36 (7.84. 10.89)** | **4.51** | **8.31** | **5.86** |
