# Supplementary material for: How health systems factors, socioeconomic status, and care-seeking practices influence the cost of rotavirus-related diarrhea care in Mukuru informal settlements, Kenya
Source: Front Public Health. 2026 Jul 24;14:1828318. doi: 10.3389/fpubh.2026.1828318 (PMC13449940; doi:10.3389/fpubh.2026.1828318)
Supplement: Supplementary file 4 [file Table_4.docx]

**Supplementary Table S4: Cost of illness based on health facilities visited**

| **COST CATEGORIES** | **Lady of Nazareth Mukuru Njenga n=5**  **Public** | | | | **MCC Mukuru Health Centre n=32**  **Public** | | | | **MMM Medical Missionaries of Mary n=42**  **Private** | | | | **NMS Mukuru Reuben Health Centre n=21**  **Public** | | | |
| --- | --- | --- | --- | --- | --- | --- | --- | --- | --- | --- | --- | --- | --- | --- | --- | --- |
| **Direct Medical Cost** | Mean [95% CI] | SD | Median | IQR | Mean [95% CI] | SD | Median | IQR | Mean [95% CI] | SD | Median | IQR | Mean [95% CI] | SD | Median | IQR |
| Expenditure at other facilities | 0.15 (0.00, 0.49) | 0.27 | 0.00 | 0.15 | 2.7 (0.00, 7.6) | 13.64 | 0.00 | 0.62 | 1.77 (0.88, 2.65) | 2.83 | 0.12 | 2.13 | 0.49 (0.04, 0.94) | 0.99 | 0.00 | 0.77 |
| Expenditure at the clinic (drugs, registration,) | 0.00 (0.00,0.00) | 2.50 | 1.62 | 1.08 | 0.00 (0.00,0.00) | 0.00 | 0.00 | 0.00 | 2.29 (1.77, 2.82) | 1.68 | 1.93 | 2.32 | 0.00 (0.00,0.00) | 0.00 | 0.00 | 0.00 |
| Prescription Drugs (Purchased Outside Clinics) | 2.41 (0.00, 5.51) | 2.50 | 1.62 | 1.08 | 3.29 (2.55 ,4.04) | 2.06 | 3.48 | 2.71 | 1.80 (1.16, 2.45) | 2.06 | 0.46 | 2.94 | 3.55 (2.64, 4.46) | 2.00 | 2.94 | 0.93 |
| **Direct Non-Medical Cost** |  |  |  |  |  |  |  |  |  |  |  |  |  |  |  |  |
| Transportation | 0 (0,0) | 0.03 | 0.00 | 0.00 | 0.07 (0.00, 0.14) | 0.21 | 0.00 | 0.00 | 0.25 (0.00, 0.55) | 0.99 | 0.00 | 0.00 | 0.00 (0.00,0.00) | 0.00 | 0.00 | 0.00 |
| Other costs (meals, airtime, etc) | 0.02 (0 , 0.06) | 0.03 | 0.00 | 0.00 | 0.16 (0.06, 0.25) | 0.26 | 0.15 | 0.15 | 0.12 (0.05, 0.19) | 0.22 | 0.00 | 0.23 | 0.09 (0.00, 0.22) | 0.28 | 0.00 | 0.00 |
| **Indirect Costs** |  |  |  |  |  |  |  |  |  |  |  |  |  |  |  |  |
| Work absence/leisure time lost | 3.49 (2.27, 4.72) | 0.99 | 3.72 | 1.11 | 3.26 (2.52, 3.94) | 1.89 | 2.97 | 1.21 | 3.47 (3.00, 3.94) | 1.51 | 3.50 | 2.32 | 2.83  (2.25, 3.40) | 1.27 | 2.60 | 1.49 |
| **Total** | **6.08  (2.15, 10.00)** | **3.16** | **4.57** | **1.32** | **9.48 (4.73, 14.22)** | **13.16** | **6.87** | **4.34** | **9.70 (8.18, 11.21)** | **4.87** | **8.81** | **6.84** | **6.99 (5.73, 8.25)** | **2.76** | **6.44** | **2.31** |
